# ERP and oscillatory differences in overweight/obese and normal-weight adolescents in response to food stimuli

**DOI:** 10.1186/s40337-020-00290-8

**Published:** 2020-04-07

**Authors:** Stefanie C. Biehl, Julian Keil, Eva Naumann, Jennifer Svaldi

**Affiliations:** 1 Department of Clinical Psychology and Psychotherapy, University of Tuebingen, Schleichstrasse 4, 72076 Tuebingen, Germany; 2 Department of Clinical Psychology and Psychotherapy, University of Regensburg, Universitaetsstrasse 31, 93053 Regensburg, Germany; 3 Biological Psychology, Christian-Albrechts University, Olshausenstraße 62, 24118 Kiel, Germany

**Keywords:** Overweight, Obesity, Adolescents, Food stimuli, Event-related potentials, Alpha band oscillations

## Abstract

**Background:**

Findings are mixed regarding the association of electroencephalographic (EEG) attentional bias measures and body weight, with few studies measuring food craving or intake and no study reporting oscillatory measures.

**Methods:**

EEG data were collected while 28 satiated adolescents (14 overweight/obese) viewed pictures of neutral, low-calorie food, and high-calorie food stimuli and rated their desire to eat, before having access to high-calorie snacks.

**Results:**

Unlike normal-weight adolescents, overweight/obese participants showed similar P300 amplitudes for high- and low-calorie food, and strongest event-related alpha band desynchronization for low-calorie stimuli. P300 amplitudes and state craving for low-calorie food furthermore predicted snack intake in this group.

**Conclusions:**

The current research focus in overweight/obesity might need to be extended to include low-calorie food. While all participants showed an attentional bias for high-calorie food, it was the processing of low-calorie food which distinguished the two weight groups on measures of neural activity and which was associated with snack food intake in the overweight/obese group.

## Plain English summary

This study tried to find out if the way the brain directs attention to food pictures is related to a person’s body weight. We therefore looked at different brain waves that indicate attention in 28 adolescents (14 were overweight or obese) while they viewed pictures of low-calorie and high-calorie food. In addition, we checked how much they craved and actually ate high-calorie food afterwards. We found that the brains of overweight/obese adolescents reacted similarly to low-calorie and high-calorie food. This was different from the normal-weight adolescents, who reacted more strongly to high-calorie food. The reaction to low-calorie food in overweight/obese adolescents was also related to the amount of snacks they ate afterwards. We think that the brain’s increased attention to low-calorie food in overweight/obese adolescents might be caused by their social environment, which constantly reminds them to watch their weight and eat low-calorie food. More studies should therefore investigate how low-calorie food is perceived and how this is related to weight and eating behaviour.

## Background

Overweight and obesity in childhood and adolescence have been recognized as a severe problem of global extent, with approximately 20 % of children and adolescents in developed countries currently classified as overweight or obese [1]. Although recent studies suggest no further increase in the rates of overweight and obesity in Western Europe and North America, numbers stagnate at a very high level with rates in Eastern Europe still increasing [2]. One of the issues frequently addressed in this context is the omnipresence of highly palatable food. In fact, experimental studies show that the mere presence of food increases food intake (see [3] for a review) and that several additional factors such as assumptions about caloric content and nutritive qualities [4, 5], portion size, sight, and smell of food [3, 6] influence food consumption. Notably though, not all children are equally susceptible to the “obesogenic” environment and recent evidence suggests that personality and cognitive factors could play a crucial role in the regulation of hedonic food intake [7, 8].

Incentive-sensitization theory provides an important framework for understanding the hedonic and motivational processes involved in the consumption of food [9, 10]. This theory posits that individual differences in mesolimbic dopaminergic functioning influence the experience of reward from food and thus its perceived hedonic value. The repeated experience of reward from the consumption of food sensitizes the individual to stimuli associated with food, thereby increasing the incentive salience of food cues. This heightened food cue reactivity then triggers attentional biases and food cravings and, consequently, increased food intake.

Predictions of this model have since been confirmed in adult populations. Functional magnetic resonance imaging (fMRI) studies investigated hypotheses regarding the hedonic and motivational value of food and found increased reward-related brain activation to high-calorie food cues in obese participants [11, 12]. While it is uncertain if these differences are cause or consequence of obesity, another study found that they moreover correlated with less successful weight loss [13]. Furthermore, reward-related functional connectivity was associated with food cravings, both in resting state and when viewing high-calorie stimuli [14, 15], and these cravings were more effortful to regulate with increasing weight [16].

Methodologies with higher temporal resolution also confirmed the predictions of the incentive salience theory. As such, eye tracking studies demonstrated that relative to normal-weight individuals, overweight/obese participants displayed a tendency for larger attentional biases [17] and greater initial attention allocation [18] to high-calorie food. Electroencephalographic (EEG) studies focused on two event-related potentials (ERPs) to examine attentional biases to food cues: The P300, a positive deflection roughly 300 ms post stimulus onset which is thought to be amplified by increased attention allocation to certain stimulus classes or motivationally salient stimuli [19, 20], and the Late Positive Potential (LPP), a slow positive wave with a centro-parietal topography starting roughly 400 ms post stimulus onset [21]. LPP amplitude has been linked to deliberate processing and strategic attention allocation and is known to be increased for emotionally salient stimuli [22–24].

In adults, both P300 and LPP amplitudes were found to be sensitive to the motivational salience of food stimuli, with higher amplitudes for food than for control pictures [25]. In line with incentive-sensitization theory, amplitudes were also increased in hungry as opposed to satiated participants [25, 26], and when high-calorie food was available versus unavailable [27]. On the trait-level, amplitudes were higher in participants who reported increased food intake in response to appetizing food stimuli (external eaters) or in response to negative emotional states (emotional eaters) [28, 29]. Several studies also investigated food craving as well as food intake as observable consequences of the attentional bias posited by incentive-sensitization theory, and P300 but not LPP amplitudes were found to correlate positively with self-reported craving [30] and subjective hunger levels in response to pictures of high-calorie food as well as with subsequent food intake [26].

In contrast, empirical evidence for the incentive salience theory in children and adolescents is less clear: fMRI studies found heightened food cue reactivity in satiated obese compared to normal-weight children [31], but also increased activation in inhibitory brain areas [31, 32]. However, when instructed to regulate food cravings, these areas showed decreased activation in heavier children [33]. Comparable to EEG findings in adults, both P300 and LPP amplitudes were found to be higher for high-calorie food cues, with higher LPP amplitudes in emotional eaters [34]. However, P300 amplitudes for mixed low- and high-calorie food cues were comparable for obese and normal-weight adolescents [35].

A change in time-frequency resolved power, namely event-related desynchronization (ERD) in the EEG alpha band [36–38] is an additional measure of the attentional and semantic processing of stimuli. An alpha band power decrease in response to a stimulus allows for more in-depth processing of the presented information by providing access to the individual’s semantic knowledge base [36]. While alpha band power changes have been investigated in some clinical samples (e.g. [39]), this has not been the case for overweight or obese participants. However, given the heightened food cue sensitivity in this population, it could be speculated that alpha band-controlled access to food-related semantic knowledge might be altered in overweight and obese participants when compared to a normal-weight sample.

In summary, there is substantial support for altered food cue responsiveness in overweight/obese participants and several studies with adult samples have investigated the association of food-related attentional biases and body weight. However, few studies also measured subjective food craving or food intake and studies with child or adolescent samples are even scarcer. To our knowledge, no study to date has attempted to connect theory-derived experimental and real life predictions by reporting the assessment of all three constructs – attentional bias, state food craving, and calorie intake – in the same adolescent sample, or to explore food cue-related neural activity with ERPs and ERD. Our study therefore strived to differentiate between low- and high-calorie foods and to replicate the previously reported high-calorie food bias in satiated adolescent participants. We further hypothesized that overweight and obese adolescents would show a stronger attentional bias for high-calorie food than normal-weight controls – as evidenced by higher P300 amplitudes – and that this attentional bias would be associated with higher self-reported state food cravings and higher subsequent snack intake. In addition, overweight/obese participants should show altered ERD in response to food stimuli, representing differences in accessing semantic knowledge upon confrontation with these cues.

## Methods

Overweight/obese (OW/OB) adolescents and a matched normal-weight (NW) control group between the ages of 9 and 16 years were recruited via newspaper articles, email announcements, and brochures handed to local paediatric and general practitioner practices. Inclusion criteria were an age- and gender-specific body mass index (BMI) percentile [40] ≥ 90 (OW/OB group) or ≤ 80 (NW group). Exclusion criteria were a history of seizures, compensatory weight control behaviour, psychotic symptoms, suicidality, medical conditions influencing weight and eating behaviour, and food allergies. After a diagnostic screening by phone, potential participants and a parent or guardian were invited for an in-depth diagnostic assessment. Both the adolescent and the parent or guardian gave their informed consent after the study was explained to them. Participants were informed that the exact hypotheses guiding the study could only be revealed afterwards to avoid biasing the results. All study procedures were in accordance with the Declaration of Helsinki [41] and approved by the local Ethics Committee (project number 557/2015B01).

Forty-three adolescents participated in the study. To rule out severe mental health problems, the *Diagnostic Interview for Mental Disorders in Children and Adolescents* (*Kinder-DIPS*) was administered to both the participant and the parent or guardian [42], and eating pathology was assessed by means of the diagnostic items of the *Child Eating Disorder Examination* (ChEDE [43]) as well as the *Child Eating Disorder Examination-Questionnaire (ChEDE-Q* [44]*)*. In addition, all participants were measured and weighed in light clothing. EEG appointments were scheduled on a different day, usually within 2 weeks of the diagnostic assessment. To standardize hunger levels, all participants were offered an ad libitum meal before the EEG appointment. Three EEG data sets could not be analysed due to technical difficulties during data collection. Data of 12 participants (six OB/OW) had to be excluded due to excessive noise or movement artefacts, which is comparable to other EEG studies with children and adolescents [45, 46]. The final sample therefore consisted of 28 adolescents, 14 in each group. The two groups were comparable in gender distribution and mean age, but significantly different in BMI percentile standard deviation scores (SDS) and ChEDE-Q global score as well as scores on the ChEDE-Q weight concern and shape concern subscales (see Table 1).

**Table 1:** Descriptive data for the overweight/obese group and the matched normal-weight control group

	Overweight/obese	Normal-weight
*n* (girls)	14 (*7*)	14 (*5*)
Age	12.6 (*1.5*)	13.5 (*1.7*)
BMI percentile SDS	2.2 (*0.44*)^b^	−0.25 (*0.81*)^b^
ChEDE-Q		
*Global*	1.6 (*1.1*)^a^	0.5 (*0.8*)^a^
*Restraint*	1.2 (*1.5*)	0.4 (*1.3*)
*Eating Concern*	1.0 (*1.3*)	0.4 (*0.6*)
*Shape Concern*	2.3 (*1.4*)^a^	0.7 (*0.9*)^a^
*Weight Concern*	2.1 (*1.1*)^b^	0.5 (*0.7*)^b^

*Note. Means and SDs (in parentheses) for age, BMI percentile SDS, and eating pathology on the Child Eating Disorder Examination Questionnaire (ChEDE-Q) for the two groups.*^*a*^*marks significant differences ≤ .01,*^*b*^*marks significant differences ≤ .001*

Participants were presented with pictures of food and office supplies in random order. Food pictures were taken from the food-pics database [47], and consisted of 30 high-calorie and 30 low-calorie food items. Food images were chosen to be comparable for contrast, complexity, spatial frequency, and available palatability rating data. Neutral images consisted of 30 pictures of office supplies that were similar in form, colour, and complexity [30]. Luminance for all food and office pictures was adjusted using a modified script from the SHINE toolbox in Matlab (The MathWorks, Natick, MA) [48]. The size of all pictures was 1024 by 768 pixels. Each picture was shown once, leading to a total of 90 trials.

Participants were seated in a sound-attenuated booth with controlled lighting, 60 cm from the screen. After three practice trials to familiarize participants with the experimental design, the study assistant left the booth and picture presentation was started. In addition to the food and office supplies, participants completed a similar task viewing emotional pictures. Results for these pictures are beyond the scope of this article and are therefore not reported here. None of the emotional pictures contained food.

**Fig. 1:**
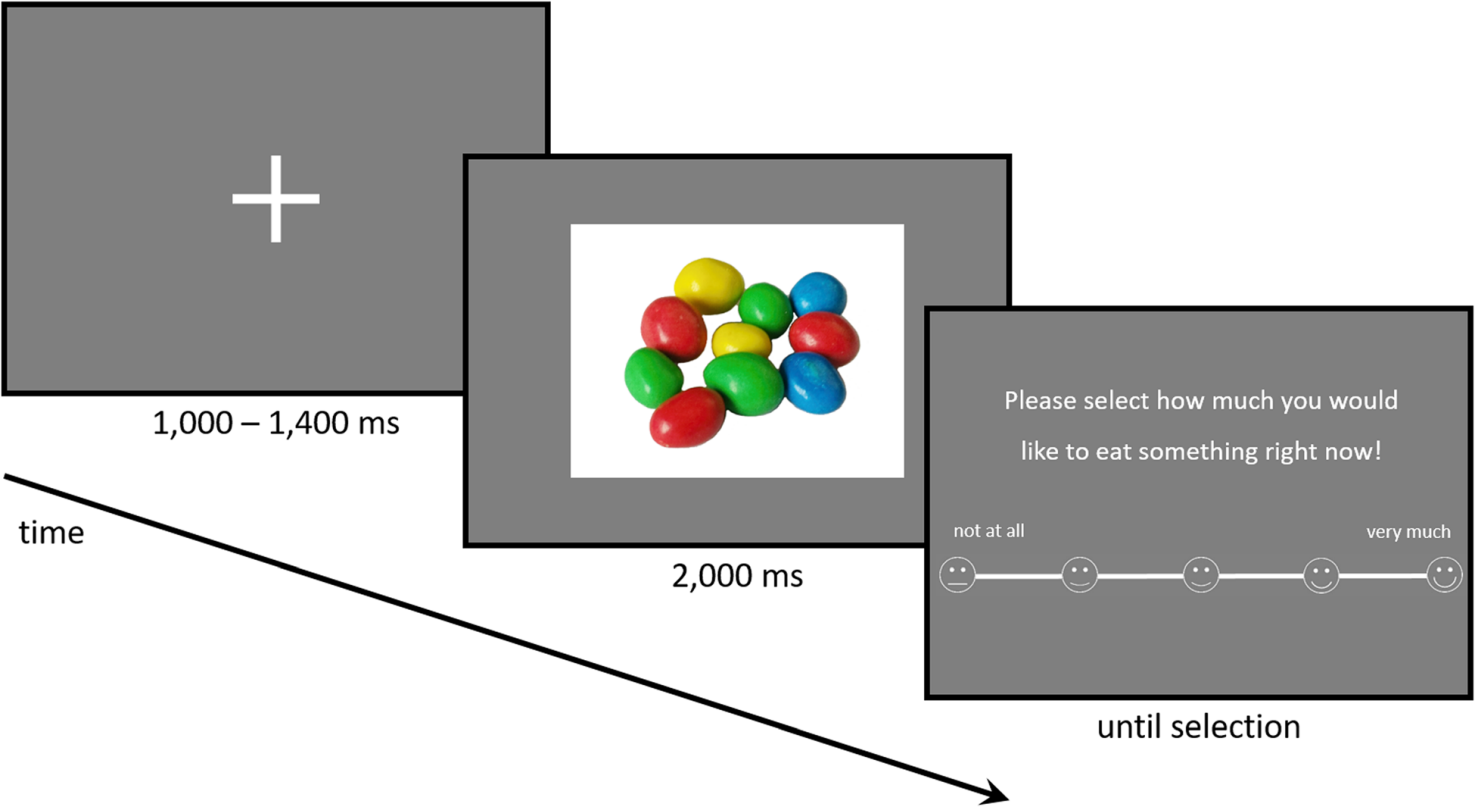
Time course of one trial, high-calorie condition, with the baseline consisting of 1000–1400 ms presentation of a fixation cross, 2000 ms active picture viewing followed by the participants’ response. The next trial started with the ISI after the response

All pictures were displayed in the center of the screen on neutral grey background in random order for 2000 ms with the interstimulus interval (ISI) jittered to last between 1000 ms and 1400 ms to provide an adequate active viewing and baseline duration for EEG data analysis. After each picture, a rating of current craving was collected on a continuous scale ranging from 1 (lowest rating) to 9 (highest rating) (based on 33). Participants were instructed to look at all pictures attentively and subsequently rate how much they would like to eat something at that very moment (see Fig. 1 for a visualization on the time course). Participants reported the ratings using the right index finger and a trackball mouse. It was stressed that there were no wrong answers and that the objective of the study was to simply collect participants’ perception of craving at a given point in time. Short breaks were inserted every 30 trials to avoid fatigue.

After the EEG experiment, the elastic cap holding the electrodes was removed and participants were led to another room with a snack buffet consisting of ten different snack foods with a total caloric value of approximately 14,600 kcal. Participants were told that the study was looking at which foods they liked best and that they could eat as much as they liked. They were only asked to try every item at least once and fill in a rating of preference for the different snacks. Participants were then left alone with the snack buffet for 20 min. All snacks were weighed before and afterwards to calculate the exact amount of consumed calories and macronutrients.

EEG data were collected from 64 Ag/AgCl active electrodes placed according to the extended 10–20 system using actiCap (Brain Products GmbH, Gilching, Germany). Data were recorded in relation to a midline reference electrode placed at Cz, with a sampling rate of 500 Hz. Four additional passive electrodes were placed above and below the right eye as well as on both outer canthi to monitor eye movement. Data were analysed according to current best practice recommendations for electrophysiological data [49] using FieldTrip in Matlab [50]: Data were segmented into three-second epochs around picture onset. Then, slow drifts in the EEG data were removed by de-trending, and a 50 Hz discrete Fourier transform filter was used to remove power line noise. To examine the unbiased EEG-signal, activity from each electrode was referenced to the average of all recorded electrodes. Subsequently, EEG data were visually inspected for movement and technical artefacts. Data sets in which more than 10% of the electrodes were affected by an excessive amount of artefacts were discarded. Eye movement artefacts were identified using independent component analysis (ICA, fastica algorithm) [51] and respective artefacts removed after visual inspection. Finally, noisy electrodes were replaced by the average of three neighbouring electrodes. For each condition, an average of 20 trials per participant could be analysed (neutral: mean = 20.4, low-calorie: mean = 20.4, high-calorie: mean = 20.1). There were slightly more usable trials for low-calorie stimuli in the OW/OB group (mean = 21.6) than in the NW group (mean = 19.1; *p* = .04), but when included as a covariate, this did not affect the significant interaction effects reported below.

For the analysis of changes in event-related potentials (ERPs), slow fluctuations and high-frequency noise were filtered out of the EEG data using a 0.1 Hz high-pass filter (Butterworth, 2nd order, 2-pass) and a 30 Hz low-pass filter (Butterworth, 4th order, 2-pass) and the average of the baseline interval (− 500 to − 100 ms prior to the stimulus onset) was subtracted from the single trial data. According to our hypothesis, we were interested in late positive ERP deflections (e.g. P300, LPP). To identify these ERP components, the EEG data were pooled across subjects and the electrodes with the largest positive peaks across all participants and conditions were identified (electrodes PO7/PO8) (see “Collapsed Localizers” in [52]). EEG data were subsequently split according to the conditions and averaged to ERPs across these electrodes within each participant. ERPs were then examined for periods of significant interaction of the between-subjects factors Group (OW/OB and NW) and the within-subjects factor Condition (neutral, low-calorie, high-calorie) in the time window from stimulus onset (0 ms) to 1000 ms using a repeated measures analysis of variance (ANOVA) at each time-point [39, 53–55]. To account for alpha error accumulation due to multiple testing, an effect was only defined as significant if it was below *p* < .05 for at least 10 ms. [56, 57]

To estimate changes in time-frequency -resolved power, single trial EEG data were transformed into the time-frequency domain using a multitaper approach [58]. Time-frequency-resolved power (1–45 Hz, 1 Hz resolution, DPSS tapers) was calculated in steps of 10 ms (window size = 3 cycles per frequency) and the average of the baseline interval (− 500 to − 100 ms prior to the stimulus onset) was removed from the single trial data (so-called relative change). Similar to the analysis of ERPs, time-frequency power was split according to the conditions and averaged for the electrode pair PO7/PO8. It was then examined for periods of significant interaction of the between-subjects factors Group (OW/OB and NW) and the within-subjects factor Condition (neutral, low-calorie, high-calorie) in the time window from stimulus onset (0 ms) to 1000 ms using a repeated measures ANOVA [59]. To account for alpha error accumulation due to multiple testing, 3dClustSim (http://afni.nimh.nih.gov/afni/) [60, 61] was used to estimate the probability of false positives. Accordingly, an effect was only defined as significant if it was below *p* < .05 in at least 34 connected time-frequency windows.

For further analyses, ERP amplitudes and time-frequency power for the significant time windows identified in the ANOVAs were exported, averaged for each participant, and further analysed using IBM® SPSS® Statistics 24. Mean ERP amplitudes, time-frequency power, and also the subjective craving ratings were entered into mixed model analyses of variance (ANOVAs) with the between-subjects factor Group (OW/OB and NW) and the within-subjects factor Condition (neutral, low-calorie, high-calorie). Values of *p* < .05 were considered significant.

## Results

### Craving data

Craving ratings showed a significant main effect of Condition (*F*_(2, 52)_ = 56.91, *p* < .001, η_p_^2^ = .69; see Table 2 for means [*M*s] and standard deviations [*SD*s]). Post-hoc t-tests conducted separately for Condition showed that across all participants, high-calorie food led to significantly higher mean craving ratings than low-calorie food and neutral items, which had the lowest ratings and were also significantly different from low-calorie food (all *p*-values < .001). There was no main effect of Group (*F*_(1, 26)_ = 2.09, *p* = .16, η_p_^2^ = .07) and no interaction of Group and Condition (*F*_(2, 52)_ = 0.19, *p* = .73, η_p_^2^ = .01). However, the main effect of Group became marginally significant when all participants were analysed (*F*_(1, 41)_ = 4.12, *p* = .049, η_p_^2^ = .09), with the NW group giving slightly higher overall ratings than the OW group.

**Table 2:** Behavioral and EEG data for the overweight/obese group and the matched normal-weight control group

	Mean subjective craving		
	*Neutral*	*Low-calorie*	*High-calorie*
Overweight/obese	1.9 (*1.1*)	4.2 (*2.0*)	5.0 (*2.7*)
Normal weight	2.5 (*1.3*)	5.1 (*1.3*)	5.9 (*1.5*)
	P300 amplitude (in μV)		
	*Neutral*	*Low-calorie*	*High-calorie*
Overweight/obese	16.1 (*7.9*)	19.0 (*8.0*)	19.7 (*8.9*)
Normal weight	22.2 (*9.0*)	21.3 (*9.5*)	23.9 (*9.2*)
	Mean alpha power (relative change)		
	*Neutral*	*Low-calorie*	*High-calorie*
Overweight/obese	−.0017 (*.0051*)	−.0026 (*.0031*)	−.0011 (*.0040*)
Normal weight	−.0033 (*.0030*)	−.0011 (*.0048*)	−.0016 (*.0035*)
	Mean snack consumption		
	*Calories (kcal)*	*Fat (g)*	*Carbohydrates (g)*
Overweight/obese	637 (*415*)	29.7 (*19.3*)	80.7 (*54.6*)
Normal weight	558 (*217*)	24.8 (*11.1*)	72.4 (*29.4*)

*Note. Means and standard deviations (in parentheses) for subjective craving, P300 amplitude, alpha power, and snack consumption, by group and condition (where applicable)*

### EEG data

#### Event-related potentials

The data driven analysis of the event-related potential (ERP) data showed a contiguous significant interaction of Group and Condition for more than 10 ms in the time window of the P300 (276–328 ms).

**Fig. 2:**
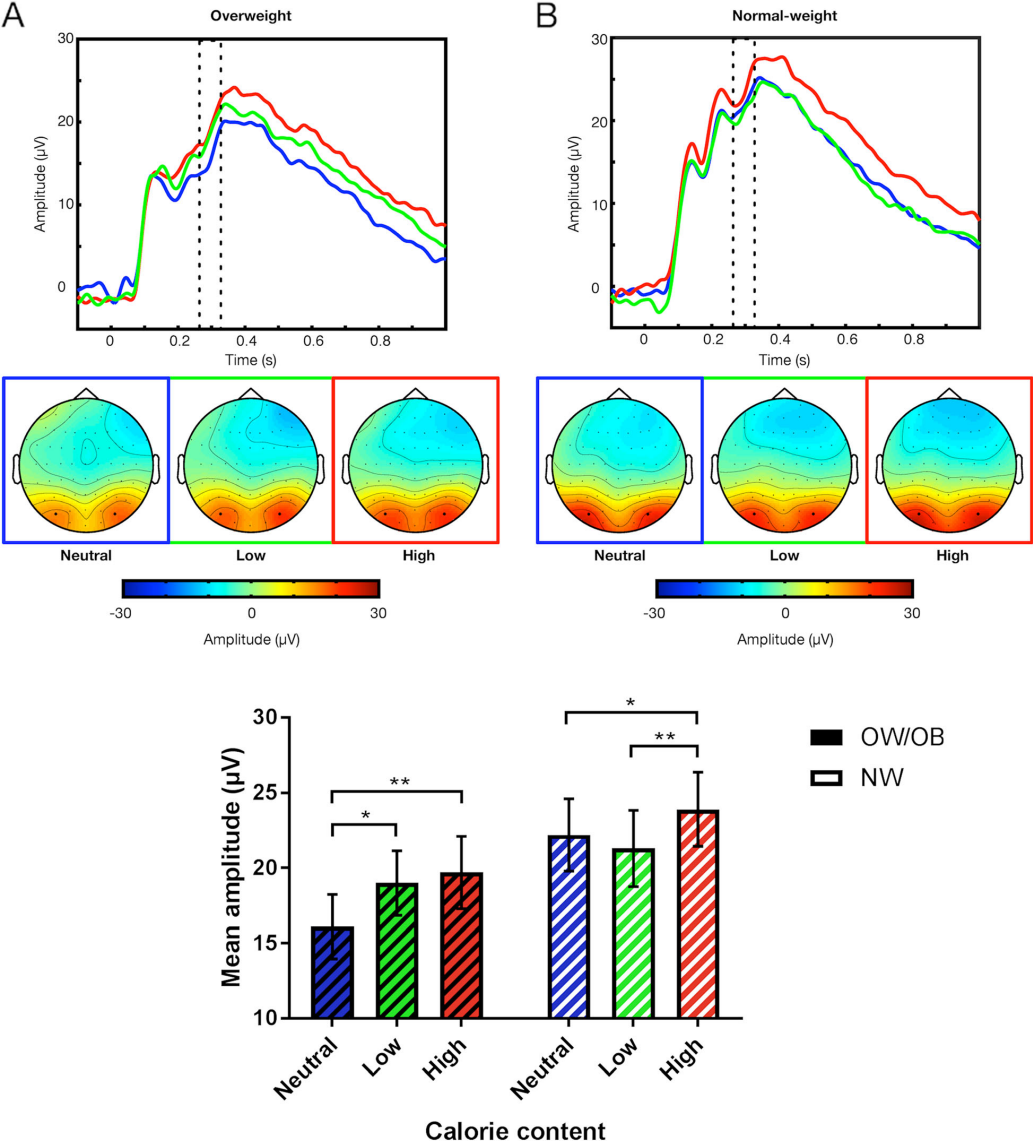
Grand-average time course for the electrode pair PO7/PO8, topography, and amplitudes by group and condition. The dotted oblong marks the time window of the significant effect; black dots mark electrodes PO7 and PO8, bars denote standard errors; * marks *p* < .05, ** marks *p* < .01

The subsequent analysis of mean individual amplitudes in this time window showed a significant main effect of Condition (*F*_(2, 52)_ = 8.53, *p* = .001, η_p_^2^ = .25; see Table 2 for *M*s and *SD*s). Post-hoc t-tests conducted separately for Condition showed that across all participants, high-calorie food led to significantly higher mean amplitudes than low-calorie food *(p* = .02) and neutral items (*p* < .001). Mean amplitudes for low-calorie food and neutral items were not significantly different (*p* = .15). There was no main effect of Group (*F*_(1, 26)_ = 1.71, *p* = .20, η_p_^2^ = .06), but the ANOVA confirmed a significant interaction of Group × Condition (*F*_(2, 52)_ = 4.56, *p* = .02, η_p_^2^ = .15; see Fig. 2). Post-hoc t-tests conducted separately for Group showed that the OW/OB group displayed significantly higher mean amplitudes for high-calorie food and for low-calorie food than for neutral items *(p* = .006 and *p* = .03, respectively), with the two food categories being not significantly different *(p* = .51). In contrast, the NW group showed significantly higher mean amplitudes for high-calorie food than for low-calorie food *(p* = .005) and for neutral items *(p* = .02), with no significant difference between low-calorie food and neutral items *(p* = .17). Post-hoc t-tests furthermore showed no significant between-group differences for amplitudes regarding the two food-categories and a trend-level difference for amplitudes to neutral stimuli *(p* = .07).

#### Time-frequency power

Exploratory analyses were carried out to examine changes in time-frequency power. The data driven analysis showed a contiguous significant interaction of Group and Condition lasting from 220 ms to 370 ms in the alpha band (8–12 Hz), indicating differences in condition-specific event-related desynchronization (ERD) between the OW/OB and the NW groups.

**Fig. 3:**
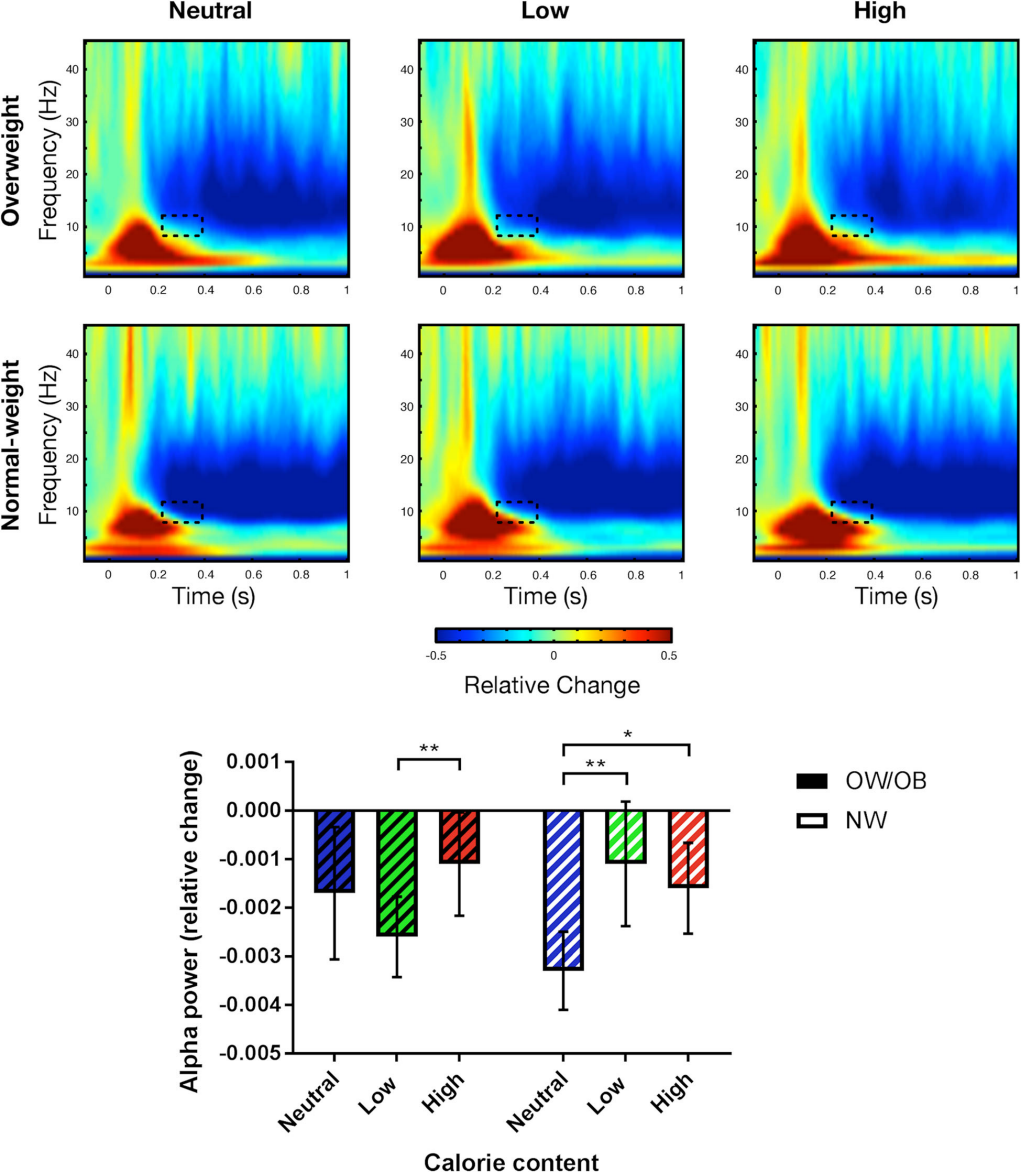
Mean time-frequency power and mean alpha ERD by group and condition. The dotted oblong marks the time window and frequency range of the significant effect; bars denote standard errors; * marks *p* < .05, ** marks *p* < .01

The subsequent analysis of mean individual power for this frequency range and time window showed a marginally significant main effect of Condition (*F*_(2, 52)_ = 3.20, *p* = .049, η_p_^2^ = .11; see Table 2 for means and standard deviations). Post-hoc t-tests conducted separately for Condition showed that across all participants, high-calorie food led to significantly less mean alpha ERD than neutral stimuli *(p* = .01). Mean alpha ERD for high-calorie and low-calorie food and for low-calorie food and neutral items was not significantly different (*p* = .30 and *p* = .18, respectively). There was no main effect of Group (*F*_(1, 26)_ = 0.20, *p* = .90, η_p_^2^ = .001), but the ANOVA confirmed a significant interaction of Group × Condition (*F*_(2, 52)_ = 5.63, *p* = .006, η_p_^2^ = .18; see Fig. 3). Post-hoc t-tests conducted separately for Group showed that the OW/OB group had significantly stronger mean alpha ERD for low-calorie food than for high-calorie food *(p* = .01). There was no significant difference for the comparison of high-calorie food and of low-calorie food with neutral items *(p* = .29 and *p* = .21, respectively; see Fig. 3). In contrast, the NW group showed significantly stronger mean alpha ERD for neutral items compared to both high-calorie food and low-calorie food *(p* = .02 and *p* = .006, respectively), with no significant difference between high-calorie food and low-calorie food *(p* = .58). Post-hoc t-tests furthermore showed no significant between-group differences for any of the three categories.

### Prediction of snack consumption

There was no significant difference in calorie and macronutrient consumption between the two groups. All participants consumed a substantial amount of the offered high-calorie food with a mean calorie intake of 598 kcal (range: 116 kcal to 1750 kcal). As the Group × Condition interaction for P300 amplitudes a was mainly driven by within-group differences for low-calorie food, mean P300 amplitudes, mean alpha ERD, and mean craving ratings for pictures of low-calorie food were correlated with calorie intake for the two groups. While there were no significant correlations for the NW group, the OW/OB group showed significant correlations for both self-reported craving (*r*_(12)_ = 0.56, *p* = .04) and mean P300 amplitudes (*r*_(12)_ = 0.61, *p* = .02) for pictures of low-calorie food: Participants with higher self-reported craving or higher P300 amplitudes subsequently consumed more snacks than participants with lower values on these two variables. A tendency for a negative association of alpha ERD and subsequent snack intake did not reach significance (*p* = .12).

## Discussion

This study intended to examine the association of three supposedly connected concepts in a single sample of adolescents: attentional bias in response to food, self-reported state craving, and increased snack food consumption.

As hypothesized, participants showed increased attention allocation to pictures of food compared to neutral pictures, as evidenced by increased P300 amplitudes for food pictures. Surprisingly, however, a preferential processing for high-calorie food was only present in normal-weight adolescents. These participants had significantly higher P300 amplitudes when viewing high-calorie food than when viewing low-calorie food or neutral items. In contrast, overweight/obese adolescents did not show different P300 amplitudes based on the caloric value of the food pictures: This group also showed a clear distinction between food and non-food pictures, but P300 amplitudes were not different for high- and low-calorie food. This is surprising, as according to incentive-sensitization theory [9, 10] low-calorie food should be associated with weaker sensitization and thus lower cue reactivity than high-calorie food.

While the general attentional bias for food versus non-food items could thus be replicated in an adolescent sample, the most important distinction between overweight/obese and normal-weight participants does not seem to be the processing of high-calorie food, but of low-calorie food. This might help explain the lack of previous findings when investigating ERP correlates of attention allocation in response to food stimuli in obese and normal-weight participants: One study with an adult sample compared high-calorie items and neutral items [25], while another study with an adolescent sample compared mixed high−/ low-calorie items and neutral items [35]. No differentiation of high-calorie and low-calorie food was made in these studies. Future studies might thus be well advised to include both high- and low-calorie food and analyse responses to these items separately.

Interestingly, the P300 group difference for low-calorie items in this study was not reflected in self-reported state craving. All adolescents reported highest state craving after viewing high-calorie items and lowest craving after viewing neutral items, with craving after viewing low-calorie items in between and no difference between the two groups. Although some studies report a negative association of state craving and BMI [62, 63], these results are in line with previous findings of no association of state craving and BMI in children and adolescents [33] and in adults [14]. In line with state craving ratings, subsequent snack intake was also not significantly different between the two groups. This is surprising given previous findings of correlations between snack intake and BMI in adults [8]. However, studies with children and adolescents also report no difference [64] or even lower snack intake in participants with higher BMI [65].

Furthermore, P300 amplitude and state craving for low-calorie food seem to be associated with subsequent snack intake (but not with each other) in the overweight/obese group. While conclusions are necessarily limited by the small sample size, this association points to the possibility of attentional biases and food cravings being two independent processes triggered by increased food cue reactivity [9, 10] with the association of food cravings and subsequent snack food intake possibly mediated by inhibitory control deficits. As previous research with a healthy adult sample found interactive effects of approach bias and inhibitory control on snack food intake [66], future studies with child and adolescent samples might benefit from directly measuring inhibitory control, while also including a measure of approach bias. In contrast, the association of food cravings and subsequent snack food intake could also be reflective of an external eating style. This would be in line with studies finding increased snack intake after smelling or tasting high-calorie snacks [64] and slower habituation to high-calorie food [67] in overweight children. It might therefore be worthwhile to consider stratifying larger samples of overweight/obese children by their inhibitory control and external eating, and to investigate the possibility of different subtypes of overeating.

The analysis of ERD in the alpha band provides additional information about the processing of food stimuli in the two groups: Neutral stimuli led to strongest ERD in the normal-weight group, which can be explained by their less frequent appearance relative to food stimuli. However, the overweight/obese group showed strongest ERD for low-calorie stimuli. Since ERD was previously hypothesized to indicate access to the individual’s semantic knowledge base [36], one could speculate that this might point to a link of greater semantic information and low-calorie food in the overweight/obese group as might be triggered by a social environment emphasizing eating “healthy” food in order to control their weight gain. However, future studies would have to include some measure of semantic memory processing to empirically test for this possibility.

Beyond the small sample size, the present study has several other limitations. Although all participants were demonstrated the EEG’s sensitivity with regard to eye and muscle movement, and were instructed to remain as still as possible during data collection, a substantial amount of data had to be discarded due to too many noisy electrodes or movement artefacts. This leaves the possibility of a biased sample with only the less active participants providing usable data. Since there seems to be some concurrence of overeating and attention-deficit/hyperactivity disorder [68], this might have particularly affected overweight/obese adolescents with pathological eating behaviour. However, the number of excluded participants was the same for both groups, indicating that data loss did not disproportionately affect overweight/obese participants. Participants might also have employed different viewing strategies to avoid processing of some of the presented pictures. Future studies might therefore benefit from including eye tracking to ensure participants’ compliance with the instructions. Alternatively, it might be useful to include an additional task that is focused on the presented pictures, although this might decrease the observable effects.

Furthermore, it is not possible to establish causality with the current cross-sectional design. Adolescents might be overweight or obese because of an inherited attraction to food irrespective of its calorie content, or low-calorie food might have gained increased importance for these participants because of social and/or familial pressure to consume these foods in an attempt to counteract their overweight or obesity. While the ERP data do not favour one of these possibilities over the other, the analysis of alpha band power actually provides some support for the latter: In the overweight/obese group, ERD was most pronounced in response to low-calorie food, which could indicate a more distinct activation of semantic knowledge [36] in response to these stimuli, as would be caused by previous experiences.

In terms of the clinical applicability of these results, giving recommendations for clinical practice is challenging. While overweight and obesity in adolescence are associated with future morbidity and mortality [69] and thus clearly need to be addressed early on, stressing the importance of consuming low-calorie food might actually lead to paradoxical effects and cause adolescents to eat more. As can be seen on the respective ChEDE-Q subscales, shape and weight concerns were increased in our overweight/obese group, which is characteristic for individuals with eating pathologies (see [70] for a review). It could therefore be speculated that seeing low-calorie food might have intensified these concerns in our overweight/obese participants, which could then have triggered increased craving and attentional bias – a possible mechanism already suggested in Williamson et al.’s influential integrated cognitive-behavioural theory of eating disorders [71]. In clinical practice, it might therefore be worthwhile to move away from the focus on nutrition and also refrain from dividing food into healthy and unhealthy or low-calorie and high-calorie categories to further weight-loss. Instead, it might be worthwhile to attempt interventions that focus on shape- and weight-related issues, e.g. mirror exposure [70], or even more unspecific approaches like mindful eating [72].

## Conclusion

To conclude, this is the first study to date to combine data collection for theory-derived cognitive and real life predictions of the incentive sensitization model by assessing attentional bias, state food craving, and snack intake in the same sample while also investigating alpha band power as an informative new measure. Our data indicate that the current research focus in children and adolescents might need to be extended to the investigation of low-calorie food. While all participants showed an attentional bias for high-calorie food, it was the processing of low-calorie food that distinguished the two weight groups on both ERP and ERD measures, and predicted snack food intake in the overweight/obese group. In the future, the assessment of attentional bias to and state craving for low-calorie food in children and adolescents might thus facilitate the creation of more individualized approaches to the early prevention and treatment of overweight and obesity.

## Data Availability

The datasets used and/or analysed during the current study are available from the corresponding author on reasonable request.

## Abbreviations

ANOVA: Analysis of variance
BMI: Body mass index
ChEDE (-Q): Child Eating Disorder Examination (−Questionnaire)
EEG: Electroencephalogram
ERD: Event-related desynchronization
ERP: Event-related potential
fMRI: Functional magnetic resonance imaging
ICA: Independent component analysis
ISI: Interstimulus interval
LPP: Late Positive Potential
M: Mean
NW: Normal-weight
OW/OB: Overweight/obese
SD: Standard deviation
SDS: Standard deviation score
TFR: Time-frequency response
